# Metabolic Dysfunction-Associated Steatotic Liver Disease and the Risk of Chronic Periodontitis: A Nationwide Cohort Study

**DOI:** 10.3390/nu17010125

**Published:** 2024-12-31

**Authors:** Bo-Kyung Shine, Minkook Son, Sang Yi Moon, Seong-Ho Han

**Affiliations:** 1 Department of Family Medicine, College of Medicine, Dong-A University, Busan 49201, Republic of Korea; kdwsbk@naver.com; 2 Department of Physiology, College of Medicine, Dong-A University, Busan 49201, Republic of Korea; physionet@dau.ac.kr; 3 Interdisciplinary Program, Department of Data Sciences Convergence, Dong-A University, Busan 49201, Republic of Korea; 4 Division of Gastroenterology and Hepatology, Department of Internal Medicine, College of Medicine, Dong-A University, Busan 49201, Republic of Korea

**Keywords:** chronic periodontitis, SLD, MASLD, MetALD, fatty liver

## Abstract

**Background:** Chronic periodontitis (CP) and metabolic dysfunction-associated steatotic liver disease (MASLD) have emerged as interconnected conditions with shared mechanisms, such as systemic inflammation and metabolic dysregulation. However, the risk of CP in the newly classified subgroups of steatotic liver disease (SLD), including MASLD and metabolic alcohol-associated liver disease (MetALD), has not been extensively studied. This study investigated the association between SLD subtypes and the incidence of CP in a nationwide cohort. **Methods:** This retrospective cohort study used data from the Korean National Health Insurance Service database. The study included 115,619 participants aged 40 and older who underwent health screenings between 2009 and 2010. The participants were classified into four groups: normal without risk factors, normal with risk factors, MASLD, and MetALD. The primary outcome was the incidence of CP as defined by ICD-10 codes and dental treatment records. Hazard ratios (HRs) were calculated using the Cox proportional hazards model and adjusted for demographic, clinical, and lifestyle factors. **Results:** Over a mean follow-up of 7.4 years, individuals with MASLD and MetALD had significantly higher risks of developing CP compared with the normal group without risk factors (MASLD: adjusted HR 1.14, 95% confidence interval (CI): 1.11–1.17; MetALD: adjusted HR 1.21, 95% CI: 1.15–1.27). The risk was more pronounced for severe CP, particularly for those with MetALD (adjusted HR 1.29, 95% CI: 1.22–1.36). Subgroup and sensitivity analyses confirmed these findings across the various definitions of hepatic steatosis and metabolic risk factors. **Conclusions:** This study reveals that individuals with MASLD and MetALD are at an elevated risk of developing CP, highlighting the need for integrated care strategies that address both periodontal health and metabolic liver conditions. These findings underscore the importance of periodontal health management in reducing the risk of CP among SLD populations.

## 1. Introduction

Non-alcoholic fatty liver disease (NAFLD), with a prevalence of 25.2%, is the most common form of chronic liver disease, and individuals with NAFLD may progress to end-stage liver diseases, such as cirrhosis and hepatocellular carcinoma [1,2,3]. However, the term NAFLD does not specify the pathogenesis or progression of the disease. Consequently, efforts have been made to identify a new diagnostic term that can facilitate a more nuanced understanding of the diversity of disease progression and provide an appropriate approach to diagnosis and treatment [4]. As a result of these efforts, in July 2023, a multinational group of experts from liver research associations agreed to revise the terminology and definitions for NAFLD to steatotic liver disease (SLD) [5]. The system categorizes patients with SLD as either having or lacking cardiometabolic risk factors (CMRFs) and alcohol consumption. The metabolic dysfunction-associated steatotic liver disease (MASLD) classification was defined as having a mild alcohol intake group among individuals with hepatic steatosis and at least one of the five CMRFs. A new category called metabolic alcohol-associated liver disease (MetALD) was introduced to describe individuals who consume moderate amounts of alcohol. MetALD is characterized by metabolic dysfunction and moderate alcohol consumption. Individuals with hepatic steatosis who do not meet the CMRF criteria are diagnosed with cryptogenic SLD. This updated framework aims to clarify the underlying causes of fatty liver disease and improve diagnostic accuracy [6,7].

The relationship between NAFLD and periodontitis has recently emerged as a significant area of investigation in this field of research [8,9,10,11,12]. These two diseases are frequently associated with inflammatory responses [13,14], insulin resistance [15,16], and metabolic syndrome [17,18] and have been postulated to interact with each other through these pathways. Chronic periodontitis (CP) has also been linked to various systemic conditions, including cardiovascular disease [19], diabetes [20], obesity [21], and dyslipidemia [22]. In particular, interest in the interaction between these two conditions has increased as a result of studies demonstrating that chronic periodontitis can exacerbate NAFLD progression by increasing the level of systemic inflammation [23,24]. The results of these studies suggest a potential pathogenic link between NAFLD and periodontitis, which may have more than a simple correlation. The recently proposed nomenclature for fatty liver disease, including MASLD, has yet to investigate the potential association between these new definitions and chronic periodontitis. This study examined the potential association between the recently proposed SLD and chronic periodontitis through a retrospective longitudinal analysis of Korean participants in the National Health Screening Program, spanning a period of approximately 7.4 years.

## 2. Methods

### 2.1. Study Design and Population

This study employed a retrospective population-based cohort study design utilizing data from the National Health Insurance Service–National Health Screening Cohort (NHIS-HealS) in Korea. The NHIS is a government-run agency that provides mandatory universal health insurance coverage to the Korean population. The National Health Screening System, which has been implemented by the Korean government, is applied to approximately 97% of the Korean population aged 40 years or older every two years. The remaining 3% is covered by a medical assistance program [25,26]. NHIS has established and made available a national health information database based on health screening results. This encompasses the NHIS-HealS database, which represents 10% of the screening participants. NHIS maintains a comprehensive database comprising sociodemographic information, health screening records, lifestyle questionnaires, treatment details (including hospitalizations and outpatient prescriptions), and diagnoses according to the International Classification of Diseases, 10th Revision (ICD-10) [27]. The amount of alcohol consumed was determined through a self-reported questionnaire.

**Figure 1:**
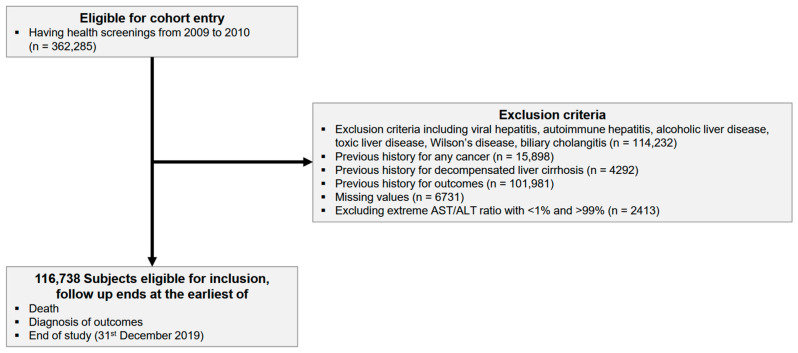
The flow of study population.

The initial cohort for this study comprised 362,285 participants from the NHIS-HealS cohort who underwent health examinations between 2009 and 2010. Of the 362,285 participants, 114,232 were excluded because they were diagnosed with viral hepatitis (B15, B16, B17, B18, and B19), autoimmune hepatitis (K754), alcoholic liver disease (K70), toxic liver disease (K71), Wilson’s disease (E830 and E831), or cholangitis (K743 and K744). Additionally, 101,981 participants diagnosed with periodontitis before the onset of chronic periodontitis, 15,898 individuals diagnosed with any type of cancer, 4929 participants with a history of decompensated cirrhosis (R17, R18, K72, I850, I864, I983, and K767), 1119 participants with alcohol use disorder, and 6731 participants with missing data were excluded from the study. Furthermore, 2413 participants with extreme AST/ALT ratios below the 1st and above the 99th percentiles were excluded. The final number of participants was 115,619. Participants were followed up until December 31, 2019, when they either died during the follow-up period or were diagnosed with the target outcome of the study or when the study ended (Figure 1).

### 2.2. Group Classification Using FLI and CMRFs: Normal (With/Without Risk), MASLD, MetALD

Individuals with a fatty liver index (FLI) of 30 or greater were classified as having SLD. The FLI is a well-established, validated, non-invasive method for identifying hepatic steatosis, and its comprehensive assessment process has been thoroughly described in the literature [28,29]. For this study, individuals with an FLI below 30 were categorized as “normal” and further subdivided into two groups based on the presence of CMRFs: normal without risk factors and normal with risk factors. The four groups analyzed were as follows: (1) normal without risk factors, (2) normal with risk factors, (3) MASLD, and (4) MetALD.

MASLD is defined by the presence of hepatic steatosis with at least one of the following five CMRFs and low alcohol consumption, defined as less than 30 g/day for men and less than 20 g/day for women: (i) a body mass index (BMI) ≥23 kg/m^2^ or waist circumference ≥90 cm for men and ≥85 cm for women; (ii) fasting serum glucose ≥100 mg/dL, type 2 diabetes mellitus, or treatment for type 2 diabetes; (iii) blood pressure ≥130/85 mmHg or treatment for hypertension; (iv) triglycerides ≥150 mg/dL or lipid-lowering therapy; (v) high-density lipoprotein (HDL) cholesterol ≤40 mg/dL in men or ≤50 mg/dL in women, or treatment with lipid-lowering therapy. MASLD is distinguished from MetALD, which involves similar metabolic risk factors but includes moderate alcohol consumption, defined as 30–60 g/day for men and 20–50 g/day for women.

### 2.3. Definitions of Covariates

Covariates were obtained from the NHIS database. Demographic variables, such as age, sex, income level (categorized into quartiles), and residence (rural, urban), were included. Medical history was assessed by identifying underlying diseases such as hypertension, diabetes, and dyslipidemia using ICD-10 codes and corresponding prescribed medications. Hypertension was identified using the ICD-10 codes I10 and I11, with criteria of admission ≥ 1 or outpatient clinic ≥ 1 visit along with any anti-hypertensive medication. Diabetes was identified using the ICD-10 codes E11, E12, E13, and E14, with criteria of admission ≥ 1 or outpatient clinic ≥ 1 visit along with any anti-diabetic medication. Dyslipidemia was identified using ICD-10 code E78, with criteria of admission ≥ 1 or outpatient clinic ≥ 1 visit along with any lipid-lowering medication. Comorbidities were evaluated using the Charlson comorbidity index (CCI) following the method described by Quan et al. [30]. Additionally, the participants’ lifestyle factors, including smoking status (never smoked, current smoker), drinking status (never drank, current drinker), weekly alcohol consumption (in grams), and exercise habits (defined as physical activity five or more times per week), were gathered through self-administered questionnaires. The BMI, blood pressure, total cholesterol, triglycerides, high-density lipoprotein, low-density lipoprotein, aspartate aminotransferase, alanine aminotransferase, gamma-glutamyl transferase, and glomerular filtration rate were obtained from health screening results.

### 2.4. Primary Outcomes

The primary outcome measure of this study was the incidence of chronic periodontitis. Chronic periodontitis was defined based on previous studies and relevant ICD-10 codes (K05: chronic periodontitis) [31]. Chronic periodontitis was diagnosed using the ICD-10 codes for dental procedures. The participants were divided into chronic periodontitis and severe chronic periodontitis groups based on their dental procedures. The chronic periodontitis group comprised patients who underwent periodontal treatment, including subgingival cauterization (U1010), tooth extraction (U4412 and U4413), periodontal flap surgery (U1051 and U1052), bone grafting for alveolar bone defects (U1071 and U1072), and guided tissue regeneration (U1081, U1082, and U1083). The severe chronic periodontitis group comprised patients with severe periodontitis, including those who had undergone dental treatment other than subgingival cauterization.

### 2.5. Statistical Analysis

The database provided by the NHIS-HealS was analyzed using two distinct software packages: SAS (version 9.4; SAS Institute Inc., Cary, NC, USA) and R version 4.3.0 (R Foundation for Statistical Computing, Vienna, Austria). The baseline demographic and clinical characteristics of the study population were summarized as means (standard deviations) for continuous variables and as counts (percentages) for categorical variables. Multiple group comparisons of baseline characteristics were performed using one-way analysis of variance (ANOVA) for continuous variables and the chi-square test for categorical variables. The incidence of chronic periodontitis over time was evaluated using the Kaplan–Meier curve in four groups: normal without risk factors, normal with risk factors, MASLD, and MetALD. Kaplan–Meier survival curves were used to illustrate the cumulative incidence of the primary outcomes, and the log-rank test was used for comparison. The proportional hazard assumption was evaluated using the Schoenfeld residuals test, which employs the logarithm of the cumulative hazard function based on Kaplan–Meier estimates. No proportional hazard assumption violations were observed. The association between the SLD subtypes and the risk of chronic periodontitis was determined by calculating the hazard ratio (HR) with a 95% confidence interval (CI) using the Cox proportional hazards model. In constructing the multivariable-adjusted Cox proportional hazards model, various covariates were considered, including age, sex, income level, residence, CCI, hemoglobin level, glomerular filtration rate, smoking, and regular exercise status. Restricted cubic spline graphs were employed in the Cox proportional hazards model to accommodate potential non-linear relationships between continuous variables and the risk of outcomes. Statistical significance was set at *p* < 0.05.

We performed a subgroup analysis and two sensitivity analyses. The subgroup analysis stratified the primary analysis by age and sex. Sensitivity analysis was performed to assess the robustness of the findings. First, a higher FLI cutoff of ≥60 was used to define SLD. Second, the main analysis was repeated using the hepatic steatosis index (HSI) ≥36 as an alternative biomarker for hepatic steatosis.

### 2.6. Ethics Statement

The necessity for individual consent from research participants was waived for this study, as the data utilized were drawn from the NHIS-HealS. The research protocol was reviewed and approved by the Dong-A University Hospital Institutional Review Board (DAUHIRB-EXP-24-134), and the research was conducted according to the ethical standards outlined in the Declaration of Helsinki.

## 3. Results

### 3.1. Patient Characteristics

Table 1 shows the baseline characteristics of the study population. The SLD cohort comprised 33.4% (n = 38,639) of the total study population. Of this group, 30.2% (n = 34,880) had MASLD and 3.2% (n = 3759) had MetALD. While women were predominant in the normal group with risk factors, males were notably more prevalent in both the MASLD and MetALD groups, with a particularly high proportion of men in the MetALD group (93.7%). Additionally, individuals in the MASLD and MetALD groups exhibited higher proportions of hypertension (55.0% and 51.6%, respectively) and diabetes (16.6% and 15.3%, respectively) than those in the normal group without risk factors.

### 3.2. Risk of Chronic Periodontitis Across SLD Subtypes

The mean follow-up period for the 115,619 participants was 7.4 years, totaling 784,088 person-years (PY) for chronic periodontitis, with an average follow-up period of 6.78 years, and 862,450 PY for severe chronic periodontitis, with an average follow-up period of 7.46 years (see Table 2). Within the chronic periodontitis subgroups, the highest adjusted hazard ratio (aHR) was observed in the MetALD group, with an aHR of 1.21 (95% CI: 1.15–1.27), followed by the MASLD group, with an aHR of 1.14 (95% CI: 1.11–1.17). Overall, the SLD status was identified as a significant risk factor for the development of chronic periodontitis. A comparison of chronic periodontitis and severe chronic periodontitis revealed that the aHR was consistently higher in all subgroups, with the highest risk in the MetALD group (aHR 1.29, 95% CI: 1.22–1.36). This suggests that high levels of alcohol consumption along with CMRFs may contribute to an increased risk of severe chronic periodontitis in the SLD population, particularly among individuals with MetALD and MASLD (Table 2).

**Figure 2:**
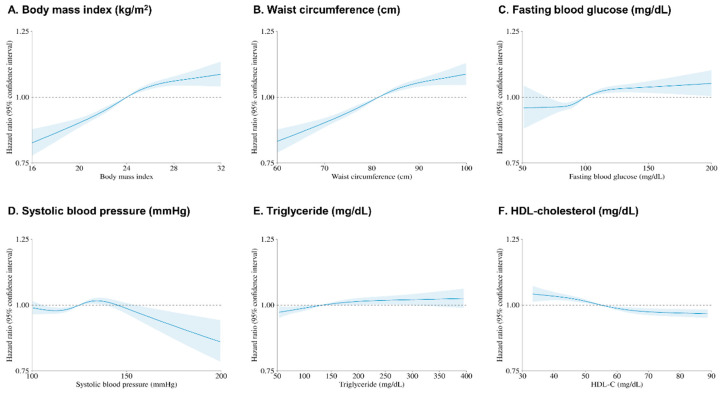
Restricted cubic spline of hazard ratio with 95% confidence intervals for chronic periodontitis. The model was adjusted for age, sex, income level, residence, Charlson comorbidity index, hemoglobin level, glomerular filtration rate, and smoking and regular exercise status.

The cumulative incidence curves in Figure 2 depict the risk of developing chronic periodontitis (Panel A) and severe chronic periodontitis (Panel B) over a 10-year follow-up period stratified by SLD subgroups (no SLD without risk factors, no SLD with risk factors, MASLD, and MetALD). The incidence of both chronic and severe chronic periodontitis was significantly higher in individuals with MASLD and MetALD than in those without SLD (*p* < 0.0001).

**Figure 3:**
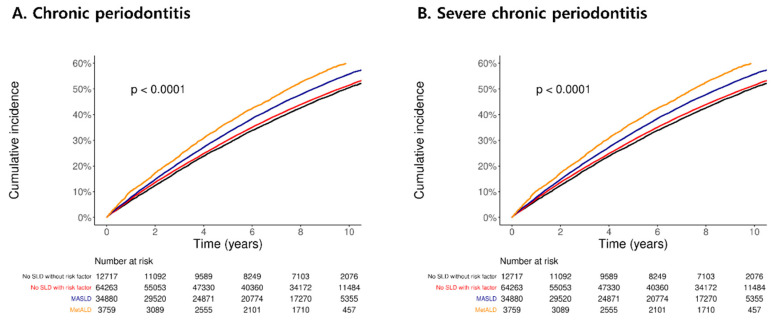
Kaplan–Meier curve for the association between SLD and chronic periodontitis.

Figure 3 shows the HR and 95% CI of the incidence of chronic periodontitis with respect to various metabolic factors. The analysis utilized restricted cubic splines in a Cox proportional hazards model, adjusted for multiple covariates. Individuals with elevated metabolic risk factors, such as higher BMI and waist circumference, demonstrated a lower probability of remaining disease-free. Conversely, Panel F shows that higher HDL cholesterol levels were associated with a reduced risk of chronic periodontitis, indicating a potential protective effect.

### 3.3. Statistical Analyses

**Figure 4:**
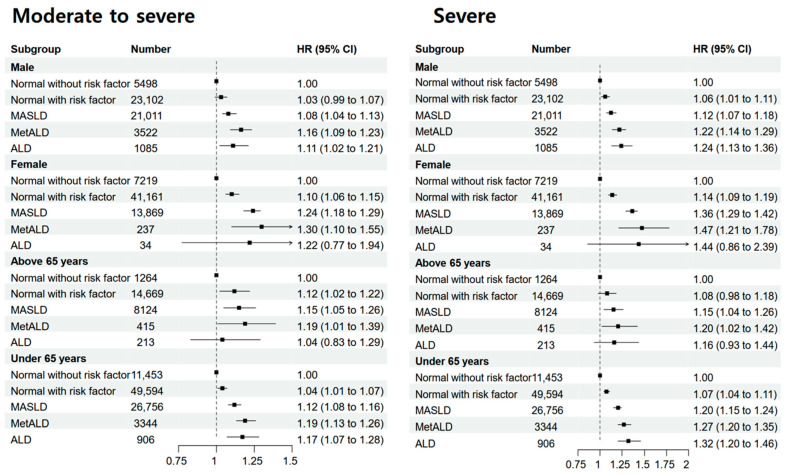
Subgroup analysis according to sex and age. The model was adjusted for age, sex, income level, residence, Charlson comorbidity index, hemoglobin level, glomerular filtration rate, and smoking and regular exercise status.

Our findings (Figure 4) indicate that individuals in the SLD subgroups (MASLD and MetALD) had a higher risk of developing both chronic and severe chronic periodontitis than those without SLD, regardless of age or sex. The risk associated with MASLD and MetALD was particularly pronounced in women and participants aged < 65 years, with stronger associations observed in patients with severe chronic periodontitis. Among women, the risk of severe chronic periodontitis increased significantly by 47% for MetALD (aHR: 1.47, 95% CI: 1.21–1.78) and 36% for MASLD (aHR: 1.36, 95% CI: 1.29–1.42), with similar patterns observed in chronic periodontitis. Supplementary Table S1 presents a sensitivity analysis of chronic periodontitis and FLI ≥ 60. Compared with the normal risk group, MASLD and MetALD participants had significantly higher risks for both chronic and severe chronic periodontitis, with aHRs of 1.15 (95% CI: 1.11–1.20) and 1.20 (95% CI: 1.12–1.29) for MASLD and MetALD, respectively, in chronic periodontitis, and 1.25 (95% CI: 1.20–1.31) and 1.36 (95% CI: 1.25–1.47), respectively, in severe cases. Supplementary Table S2, using HSI ≥ 36, confirms these findings, reinforcing the association between hepatic steatosis and periodontitis.

## 4. Discussion

The primary clinical significance of this study was to assess the risk of developing periodontal disease across the newly proposed SLD subgroups in a large nationwide cohort of 115,619 participants followed over a nearly 7.4-year period. Our analysis evaluated the incidence of periodontal disease according to the SLD subtypes, specifically MASLD and MetALD, compared with individuals without SLD and without CMRFs. For chronic periodontitis, the aHR was 1.14 (95% CI: 1.11–1.17) for MASLD and 1.20 (95% CI: 1.13–1.26) for MetALD. For severe chronic periodontitis, MASLD had an aHR of 1.22 (95% CI: 1.18–1.26), and MetALD had an aHR of 1.28 (95% CI: 1.22–1.33). These results demonstrate a clear association between SLD subtypes and increased risk of periodontal disease, with higher alcohol consumption levels further elevating the risk.

The relationship between NAFLD and periodontal disease has been investigated for several decades. Early studies, such as those by Movin [32], Novacek et al. [33], and Anand et al. [8], conducted between the 1980s and early 2000s, examined the potential impact of periodontal disease on liver conditions, including cirrhosis. These foundational studies highlighted the possibility of an interplay between periodontal health and liver disease, laying the foundation for subsequent research. Building on these findings, Kuraji’s 2021 review introduced the concept of the oral–liver axis, which connects periodontal disease and NAFLD through shared pathways such as chronic inflammation and microbial dysbiosis [11]. In 2024, Kuraji et al. expanded on this concept through an experimental study demonstrating that periodontal disease can exacerbate liver steatosis and mitochondrial oxidative stress via oral, gut, and liver dysbiosis. Their findings also highlighted the therapeutic potential of targeting dysbiosis with agents such as nisin lantibiotic, which effectively mitigated liver dysfunction. This study provides mechanistic insights into the complex relationship between oral health and liver disease, reinforcing the importance of addressing periodontal dysbiosis to reduce systemic inflammation and metabolic complications [34]. Moreover, a meta-analysis by Aguiar in 2023 offered a quantitative assessment of the relationship between NAFLD and periodontal disease, demonstrating a 91% increased risk of periodontal disease in NAFLD patients (OR: 1.91; 95% CI: 1.21–3.02; *p* = 0.006) [35]. Additionally, a Korean cohort study reinforced this association by revealing a dose-dependent relationship between periodontitis and the risk of NAFLD, suggesting that managing periodontal health could help mitigate the risk [36]. In alignment with these findings, our study demonstrated a significant association between periodontitis and MASLD, a newly redefined term for NAFLD. These results not only correspond with previous research but also reinforce the importance of periodontal health as a modifiable factor in reducing MASLD risk.

A large body of evidence from a range of studies has substantiated the link between chronic periodontitis and metabolic diseases [11,37,38,39]. Building on this, the study analyzed data from the Korea National Health and Nutrition Examination Survey (NHANES) (1988–2014) for 4297 adults with diabetes and revealed that moderate/severe periodontitis is associated with higher risks of all-cause mortality (HR: 1.27) and cardiovascular disease (CVD)-related mortality (HR: 1.35) [19]. Periodontitis also improved mortality prediction beyond the standard risk factors, indicating its potential as a marker for residual risk [19]. Preshaw et al. explored the bidirectional relationship between chronic periodontitis and diabetes and highlighted how periodontitis worsens insulin resistance and increases systemic inflammation in patients with diabetes. Their review summarizes the evidence that periodontitis contributes to elevated levels of HbA1c and that periodontal treatment can improve glycemic control, with short-term reductions in HbA1c by 0.3–0.4% [20].

Obesity is a well-established risk factor for chronic periodontitis, primarily mediated through systemic inflammation. Elevated BMI, waist circumference, and body fat are strongly associated with increased risks of periodontitis, with inflammatory mediators such as TNF-α and IL-6 playing critical roles [21]. It provides strong epidemiological evidence linking severe periodontitis to an increased risk of atherosclerotic CVD, with possible mechanisms involving systemic inflammation and the presence of periodontal pathogens in atherothrombotic tissues [40]. Muñoz Aguilera et al. conducted a meta-analysis that revealed that individuals with moderate-to-severe periodontitis had higher blood pressure than those without, with a weighted mean difference (WMD) of 4.49 mmHg for systolic blood pressure (SBP) and 2.03 mmHg for diastolic blood pressure (DBP) (*p* < 0.00001). Additionally, severe periodontitis increased the odds of hypertension by 49% (OR 1.49, 95% CI: 1.09–2.50) [41]. In a cross-sectional study by Gomes-Filho et al., a significant association was observed between the severity of periodontitis and dyslipidemia. Among the 1011 participants, those with moderate and severe periodontitis had a 30% and 16% higher likelihood of dyslipidemia, respectively, than those without periodontitis [22]. Research investigating the correlations among the components of metabolic syndrome has established that metabolic disorders can induce systemic inflammation, thereby promoting the progression of chronic periodontitis [42,43].

The link between chronic periodontitis and alcohol consumption has been corroborated by numerous studies [44,45,46]. A meta-analysis by Pulikkotil et al. investigated the link between alcohol consumption and periodontitis; they observed a significant association where alcohol consumers had a 26% higher risk of periodontitis (OR = 1.26, 95% CI: 1.11–1.41) [47]. A prospective study involving 39,461 male healthcare professionals revealed that alcohol consumption is significantly associated with an increased risk of periodontitis. Compared with non-drinkers, men consuming alcohol had progressively higher relative risks of periodontitis, with those drinking >30 g/day having a 27% increased risk (RR: 1.27, 95% CI: 1.08–1.49) [48]. Because both MASLD and MetALD are associated with CMRFs, it appears reasonable to conclude that the increased risk of periodontal disease may be interpreted as a consequence of increased alcohol consumption. Consequently, modifying alcohol consumption habits or promoting alcohol abstinence may prove to be an effective therapeutic strategy for patients with periodontitis.

The limitations of this study were as follows. First, this study was a retrospective analysis based on claims data, which precluded the inclusion of imaging and histological information; as a result, the accuracy of the diagnoses may be compromised. Additionally, data from the NHIS may be affected by changes in the insurance system or policy, which may result in inconsistencies in the data. The age range of the analyzed patients was limited to 40 years or older, and the follow-up period for the elderly patients was relatively short, which may have influenced the results. Furthermore, as the study participants were only Koreans, inherent limitations exist in generalizing the results to other races or regions. Another limitation is the mutual association observed between periodontitis and fatty liver disease, as it remains unclear whether periodontitis precedes fatty liver disease or vice versa. This uncertainty increases the possibility of a bidirectional relationship, in which both diseases mutually influence each other, complicating the interpretation of causality [49,50]. Despite these limitations, this study has several notable strengths in its design. By utilizing a large, nationwide cohort of 115,619 participants with nearly 7.4 years of follow-up data, we ensured robust statistical power and comprehensive analysis. Furthermore, the application of sensitivity and stratification analyses, as well as competing risk models, enhanced the validity and reliability of our findings. These methodological approaches address potential biases and allow for a more nuanced understanding of the relationship between MASLD/MetALD and chronic periodontitis. This study also provides significant clinical implications. By identifying a clear association between MASLD/MetALD and chronic periodontitis, our findings highlight the need for integrated healthcare management strategies. Given these findings, implementing bidirectional screening practices could be beneficial. Specifically, patients diagnosed with periodontitis may benefit from routine metabolic disorder evaluations, while individuals with metabolic conditions should undergo periodontal health assessments. Such an approach would facilitate early detection and integrated management, ultimately improving outcomes for both metabolic and oral health conditions. These results provide a foundation for future studies aimed at developing risk prediction models and exploring interdisciplinary approaches to the management of MASLD, MetALD, and chronic periodontitis.

## 5. Conclusions

The presence of MASLD or MetALD is associated with a higher risk of developing chronic periodontitis than the absence of SLD. Individuals with MASLD or MetALD require integrated healthcare management, which should encompass not only the management of their metabolic disorders and alcohol intake but also an enhanced screening program for periodontal disease.

## Figures and Tables

**Table 1:** Baseline characteristics of study population.

Variables		Normal Without Risk Factor (n = 12,717)	Normal with Risk Factor (n = 64,263)	MASLD (n = 34,880)	MetALD (n = 3759)	*p*-Value
Sex (%)	Male	5498 (43.2)	23,102 (35.9)	21,011 (60.2)	3522 (93.7)	<0.001
	Female	7219 (56.8)	41,161 (64.1)	13,869 (39.8)	237 (6.3)	
Age (years)	Mean (SD)	54.7 (7.3)	58.6 (9.1)	58.8 (8.8)	55.1 (7.2)	<0.001
Income level (%)	1st quartile	1716 (13.5)	9715 (15.1)	4712 (13.5)	422 (11.2)	<0.001
	2nd quartile	2820 (22.2)	13,889 (21.6)	6902 (19.8)	652 (17.3)	
	3rd quartile	3449 (27.1)	18,292 (28.5)	10,220 (29.3)	1178 (31.3)	
	4th quartile	4732 (37.2)	22,367 (34.8)	13,046 (37.4)	1507 (40.1)	
Residence (%)	Rural	3813 (30.0)	22,384 (34.8)	12,273 (35.2)	1301 (34.6)	<0.001
	Urban	8904 (70.0)	41,879 (65.2)	22,607 (64.8)	2458 (65.4)	
Hypertension (%)		0 (0.0)	27,214 (42.3)	19,173 (55.0)	1939 (51.6)	<0.001
Diabetes (%)		0 (0.0)	5693 (8.9)	5784 (16.6)	577 (15.3)	<0.001
Dyslipidemia (%)		0 (0.0)	22,348 (34.8)	17,941 (51.4)	1426 (37.9)	<0.001
Charlson comorbidity index (%)	0	8370 (65.8)	33,057 (51.4)	16,886 (48.4)	2263 (60.2)	<0.001
	1	3184 (25.0)	17,734 (27.6)	9441 (27.1)	941 (25.0)	
	2	919 (7.2)	7740 (12.0)	4450 (12.8)	359 (9.6)	
	≥3	244 (1.9)	5732 (8.9)	4103 (11.8)	196 (5.2)	
Body mass index (kg/m^2^)	Mean (SD)	20.9 (1.5)	23.0 (2.2)	26.2 (2.5)	25.5 (2.4)	<0.001
Waist (cm)	Mean (SD)	72.9 (5.7)	78.1 (6.3)	88.0 (6.2)	87.9 (6.2)	<0.001
Systolic blood pressure (mmHg)	Mean (SD)	113.5 (10.7)	124.1 (15.2)	128.8 (14.9)	130.2 (14.6)	<0.001
Diastolic blood pressure (mmHg)	Mean (SD)	70.8 (7.5)	76.8 (9.9)	79.9 (9.8)	81.9 (10.0)	<0.001
Fasting blood glucose (mg/dL)	Mean (SD)	87.7 (7.4)	97.5 (20.2)	104.1 (26.3)	106.4 (27.3)	<0.001
Total cholesterol (mg/dL)	Mean (SD)	190.1 (25.4)	199.9 (37.0)	208.2 (38.4)	205.2 (36.5)	<0.001
Triglyceride (mg/dL)	Mean (SD)	81.0 (28.2)	107.2 (49.1)	187.1 (95.1)	197.5 (112.6)	<0.001
HDL cholesterol (mg/dL)	Mean (SD)	62.6 (18.2)	56.2 (21.5)	50.6 (26.2)	53.5 (16.7)	<0.001
LDL cholesterol (mg/dL)	Mean (SD)	111.5 (24.3)	122.8 (35.9)	121.6 (38.2)	113.5 (42.2)	<0.001
Aspartate aminotransferase (U/L)	Mean (SD)	23.2 (9.3)	23.4 (7.6)	26.9 (13.4)	29.5 (16.1)	<0.001
Alanine aminotransferase (U/L)	Mean (SD)	18.6 (11.2)	20.0 (9.7)	28.4 (16.7)	29.6 (20.0)	<0.001
r-glutamyl transpeptidase (U/L)	Mean (SD)	20.8 (15.0)	21.1 (12.7)	43.6 (39.9)	74.6 (65.4)	<0.001
Hemoglobin (g/dL)	Mean (SD)	13.4 (1.4)	13.4 (1.4)	14.2 (1.5)	14.9 (1.2)	<0.001
Glomerular filtration rate (mL/min/1.73 m^2^)	Mean (SD)	81.8 (29.1)	78.9 (30.5)	76.8 (32.2)	80.1 (32.2)	<0.001
Smoking (%)	Non-smoker	9331 (73.4)	50,714 (78.9)	21,913 (62.8)	1087 (28.9)	<0.001
	Ex-smoker	1539 (12.1)	7465 (11.6)	7008 (20.1)	1208 (32.1)	
	Smoker	1847 (14.5)	6084 (9.5)	5959 (17.1)	1464 (38.9)	
Alcohol drinking (%)		4540 (35.7)	18,353 (28.6)	13,952 (40.0)	3759 (100.0)	<0.001
Amount of Alcohol drinking (g/week)	Mean (SD)	36.6 (88.2)	29.5 (84.9)	33.2 (53.6)	284.7 (70.0)	<0.001
Regular exercise (%)		530 (4.2)	2840 (4.4)	1407 (4.0)	149 (4.0)	<0.001
Fatty liver index	Mean (SD)	7.3 (5.2)	14.6 (7.7)	49.7 (15.3)	56.1 (17.3)	<0.001

**Table 2:** Association between SLD and chronic periodontitis.

Group	Number	Events	Follow-Up Duration (Person-Years)	Incidence Rate (per 1000 Person-Years)	Crude HR (95% CIs, *p*-Value)	Adjusted HR (95% CIs, *p*-Value) *
Chronic periodontitis						
Normal without risk factor	12,717	6209	89,148	69.65	1 (Reference)	1 (Reference)
Normal with risk factor	64,263	31,871	440,886	72.29	1.04 (1.01–1.07, *p* < 0.007)	1.06 (1.03–1.09, *p* <0.001)
MASLD	34,880	18,717	230,485	81.21	1.17 (1.13–1.20, *p* < 0.001)	1.14 (1.11–1.17, *p* < 0.001)
MetALD	3759	2198	23,569	93.26	1.34 (1.28–1.41, *p* <0.001)	1.21 (1.15–1.27, *p* <0.001)
Severe chronic periodontitis						
Normal without risk factor	12,717	4621	98,881	46.73	1 (Reference)	1 (Reference)
Normal with risk factor	64,263	25,081	485,048	51.71	1.11 (1.07–1.14, *p* <0.001)	1.09 (1.06–1.13, *p* <0.001)
MASLD	34,880	15,507	252,477	61.42	1.32 (1.27–1.36, *p* < 0.001)	1.22 (1.18–1.26, *p* < 0.001)
MetALD	3759	1828	26,044	70.19	1.51 (1.43–1.59, *p* <0.001)	1.29 (1.22–1.36, *p* <0.001)

* The model was adjusted for age, sex, income level, residence, Charlson comorbidity index, hemoglobin level, glomerular filtration rate, and smoking and regular exercise status.

## Data Availability

The datasets generated and/or analyzed in the current study are available from the corresponding author upon reasonable request. Requests to access these datasets should be directed to B.-K.S.

## Supplementary Materials

The following supporting information can be downloaded at: https://www.mdpi.com/article/10.3390/nu17010125/s1, Table S1. Sensitivity analysis with fatty liver index over 60; Table S2. Sensitivity analysis with HSI.
