# Supplementary material for: Metabolic Dysfunction-Associated Steatotic Liver Disease and the Risk of Chronic Periodontitis: A Nationwide Cohort Study
Source: Nutrients. 2024 Dec 31;17(1):125. doi: 10.3390/nu17010125 (PMC11723414; doi:10.3390/nu17010125)
Supplement: Supplementary file 1 [file nutrients-17-00125-s001.zip › nutrients-3378051-SI-main.pdf]

Supplementary Table S1. Sensitivity analysis with Fatty liver index over 60.

Group	Number	Events	Follow-up duration (person-years)	Incidence rate (per 1000 person-years)	Crude HR (95% CIs, *p*-value)	Adjusted HR (95% CIs, *p*-value)*
**Chronic periodontitis**						
**Normal without risk factor**	12,717	6209	89,148	69.65	1 (Reference)	1 (Reference)
**Normal with risk factor**	92,939	47,274	630,735	74.95	1.08 (1.05–1.11, <0.001)	1.08 (1.06–1.11, <0.001)
**MASLD**	8483	4643	55,029	84.37	1.21 (1.17–1.26, $p <$ 0.001)	1.15 (1.11–1.20, $p <$ 0.001)
**MetALD**	1480	869	9175	94.71	1.36 (1.27–1.46, <0.001)	1.20 (1.12–1.29, <0.001)
**Severe chronic periodontitis**						
**Normal without risk factor**	12,717	4621	98,881	46.73	1 (Reference)	1 (Reference)
**Normal with risk factor**	92,939	37,755	693,546	54.44	1.17 (1.13–1.20, <0.001)	1.13 (1.09–1.16, <0.001)
**MASLD**	8483	3909	60,049	65.1	1.40 (1.34–1.46, $p <$ 0.001)	1.25 (1.20–1.31, $p <$ 0.001)
**MetALD**	1480	752	9975	75.39	1.62 (1.50–1.75, <0.001)	1.36 (1.25–1.47, <0.001)

*The model was adjusted for age, sex, income level, residence, Charlson comorbidity index, hemoglobin level, glomerular filtration rate, smoking and regular exercise status.

Supplementary Table S2. Sensitivity analysis with HSI.

Group	Number	Events	Follow-up duration (person-years)	Incidence rate (per 1000 person-years)	Crude HR (95% CIs, *p*-value)	Adjusted HR (95% CIs, *p*-value)*

**Chronic periodontitis**						
**Normal without risk factor**	12,648	6173	88,666	69.62	1 (Reference)	1 (Reference)
**Normal with risk factor**	81,087	41,048	548,928	74.78	1.07 (1.05–1.10, <0.001)	1.07 (1.05–1.10, <0.001)
**MASLD**	20,727	11,106	139,111	79.84	1.15 (1.11–1.18, $p < 0.001$)	1.16 (1.13–1.20, $p < 0.001$)
**MetALD**	1080	627	6857	91.44	1.31 (1.21–1.43, <0.001)	1.18 (1.09–1.29, <0.001)
**Severe chronic periodontitis**						
**Normal without risk factor**	12,648	4591	98,368	46.67	1 (Reference)	1 (Reference)
**Normal with risk factor**	81,087	32,724	603,593	54.22	1.17 (1.13–1.20, <0.001)	1.11 (1.08–1.15, <0.001)
**MASLD**	20,727	9159	152,400	60.10	1.29 (1.24–1.34, $p < 0.001$)	1.27 (1.22–1.32, $p < 0.001$)
**MetALD**	1080	528	7528	70.14	1.51 (1.38–1.65, <0.001)	1.32 (1.20–1.44, <0.001)

*The model was adjusted for age, sex, income level, residence, Charlson comorbidity index, hemoglobin level, glomerular filtration rate, smoking and regular exercise status.
